# Ecotoxicity of Plastics from Informal Waste Electric and Electronic Treatment and Recycling

**DOI:** 10.3390/toxics8040099

**Published:** 2020-11-08

**Authors:** Maria Angela Butturi, Simona Marinelli, Rita Gamberini, Bianca Rimini

**Affiliations:** 1 Department of Sciences and Methods for Engineering, University of Modena and Reggio Emilia, Via Amendola 2, 42122 Reggio Emilia, Italy; simona.marinelli@unimore.it (S.M.); rita.gamberini@unimore.it (R.G.); bianca.rimini@unimore.it (B.R.); 2 Interdepartmental Research Center for Industrial Research and Technology Transfer in the Field of Integrated Technologies for Sustainable Research, Efficient Energy Conversion, Energy Efficiency of Buildings, Lighting and Home Automation, University of Modena and Reggio Emilia, 42122 Reggio Emilia, Italy

**Keywords:** e-plastics, toxicity, flame retardants, informal WEEE treatment, LCA, USEtox

## Abstract

Plastic materials account for about 20% of waste electrical and electronic equipment (WEEE). The recycling of this plastic fraction is a complex issue, heavily conditioned by the content of harmful additives, such as brominated flame retardants. Thus, the management and reprocessing of WEEE plastics pose environmental and human health concerns, mainly in developing countries, where informal recycling and disposal are practiced. The objective of this study was twofold. Firstly, it aimed to investigate some of the available options described in the literature for the re-use of WEEE plastic scraps in construction materials, a promising recycling route in the developing countries. Moreover, it presents an evaluation of the impact of these available end-of-life scenarios on the environment by means of the life cycle assessment (LCA) approach. In order to consider worker health and human and ecological risks, the LCA analysis focuses on ecotoxicity more than on climate change. The LCA evaluation confirmed that the plastic re-use in the construction sector has a lower toxicity impact on the environment and human health than common landfilling and incineration practices. It also shows that the unregulated handling and dismantling activities, as well as the re-use practices, contribute significantly to the impact of WEEE plastic treatments.

## 1. Introduction

Globally, an amount approaching 50 million tons of potentially hazardous electronic waste (e-waste or WEEE-waste electrical and electronic equipment) is generated [1]. Proper collection and re-use (or refurbishing) of WEEE [2] and resource recovery are mandatory for pollution prevention and hazard reduction [3].

After the recovery of metals, which is a priority for recycling, plastics are the second most important material for recycling purposes [4]. The plastic fraction of WEEE represents, on average, about 20% in e-waste weight, depending on the device category [5]. The recycling of e-waste plastics is a complex issue from a techno-economic point of view, mainly due to the mixed material and diverse material composition, but it also poses environmental and human health issues since e-waste plastics contain potentially harmful additives [6].

One of the main and most harmful categories of WEEE plastic (WEEEP) additives includes brominated flames retardants (BFRs), used to retard ignition and prevent fire from spreading in electronic devices. These halogenated functional additives act chemically by stopping flame propagation; they must be compatible with the polymer they are added to, and must be stable during the lifespan of the plastic product [7]. This required stability causes many BFRs to be persistent, bio-accumulating, and toxic, and to be classified as persistent organic pollutants (POPs), according to the UN Stockholm Convention (http://www.pops.int/). BFRs declared as POPs are no longer allowed as additives, but the amount of e-waste deriving from formerly produced items, still circulating around the world, can cause the release of the toxic substances into the environment during the collection, transportation, handling, storage, re-processing, and disposal phases. Moreover, while the handling, re-processing, and disposal phases of WEEE plastics containing such materials are now regulated in many developed countries, mainly Europe, North America, Japan, and some Southeast Asian nations [8], imposing high-level technical standards in recycling facilities, limiting the workers’ exposure, and minimizing the environmental impact, the situation is different in low- and middle-income countries (LMICs) that are, despite the Basel Ban [9], the main destinations of uncontrolled e-waste routes [10]. Thus, mainly in LMICs, informal and unregulated e-waste dismantling, recycling, and landfilling pose environmental and human health issues [11]. In many developing countries, such as India, many African nations, and most of the nations of Latin America [12], where informal recycling still uses rudimentary methods, BFRs can enter the environment through direct diffusion from treated objects at room temperature through photo-decomposition [13]. Moreover, a relevant proportion of e-waste is landfilled without controls, producing harmful effects to the environment [14]. The other end-of-life option for e-plastics is incineration, with the aim of recovering energy; the thermal decomposition of BFRs generates dioxins and furans as well as small and large brominated species [13], which, in many developing countries, where the practice of open burning of WEEE is still common [15], are released into the environment.

The e-waste management problem has a high complexity and many critical points, combining logistics [16] and techno-economic issues with environmental and human health concerns [17], and must be tackled at a global level, promoting scientific innovation and involving policy, society, and industry [18]. However, some initiatives tailored to local needs and resources [19] can contribute to reducing the release of hazardous chemicals and materials in order to minimize their adverse impacts on human health and the environments [20].

Focusing on the plastic component of e-waste, the re-use of e-plastics as recycled aggregates (recycled plastic aggregates—RPAs) in the construction sector is considered by many researchers as one of the main recycling routes for e-plastics in developing countries [21]. Plastic is a low degradable material that has become an environmental burden. On the other hand, some of its characteristics (low permeability, low density, smooth surface, and good acid resistance) make it suitable for reuse in construction applications, such as concrete blocks, pavement concrete blocks, and lightweight concrete blocks [22]. These applications contribute to reducing the excessive mining and usage of natural resources, such as aggregate and cement, mostly where plastic recycling, or incineration for energy recovery, is not possible due to the lack of a dedicated infrastructure, like in many LMICs [23]. Moreover, in these countries, the use of plastic for building constructions can effectively respond to the need for low-cost construction materials due to growing urbanization [24].

Thus, the re-use of e-plastics containing BFRs in construction materials seems a promising pathway to reduce the environmental impacts and the human health risks due to toxic pollutants in developing countries. To investigate the impact of these recycling practices, this study compares five end-of-life scenarios, including both the common practices (open burning and landfilling) and some of the available options described in the literature for the re-use of e-plastic scraps in construction materials. We use the life cycle assessment (LCA) approach, a methodology standardized by the requirements of the ISO 14040 series, able to quantify the potential environmental impacts related to products, processes, or services, largely used to assess waste management systems [25]. In this study, the methodology is used to focus on ecotoxicity effects, in order to investigate specifically potential toxicological effects on human health and freshwater ecosystems, related both to informal e-plastic treatment processes and to different recycling practices.

## 2. Materials and Methods

The research methodology of this study comprises two main steps. The first step uses a literature review to investigate the end-of-life options for e-plastic containing BFR in countries where unregulated WEEE handling and disposal pose major environment and human health concerns; taking into account the specificity of such local economies, the focus was the re-use of mixed e-plastic as recycled components in construction materials. The second step uses the LCA methodology to investigate the environmental impacts of the informal treatment of WEEE plastics with a comparison of different end-of-life alternative scenarios.

Specifically, the literature search and the LCA study aimed at answering the following questions:

How can mixed e-plastic containing BFR as hazardous materials be re-used as recycled components in construction materials, in developing countries?

What are the environmental and health risks related to informal e-waste treatments and e-plastic re-use alternatives?

### 2.1. Literature Overview and Research Background

WEEE plastics count more than 15 different types of engineering plastics; typical e-waste plastic composition includes ABS (Acrylonitrile Butadiene Styrene) (30%), HIPS (High Impact Polystyrene) (25%), PP (polypropylene) (8%), PC (polycarbonate) (10%), PC/ABS (9%), and other mixed materials (18%) [26]. Around 3400 WEEE items contain plastic components, often composed of several polymer types. Moreover, about 25% of e-plastics are flame-retardant plastic [27].

Thus, the recycling of e-plastics is still a complex issue, both from the techno-economic and the environmental point of view. The recovery and re-manufacturing of e-plastics require a number of onerous and potentially hazardous technical steps, due to the number of plastic materials used in electronic devices, the valuable metal and element content, and toxic additives [28].

#### 2.1.1. WEEE Plastic Treatments and Recycling

**Figure 1:**
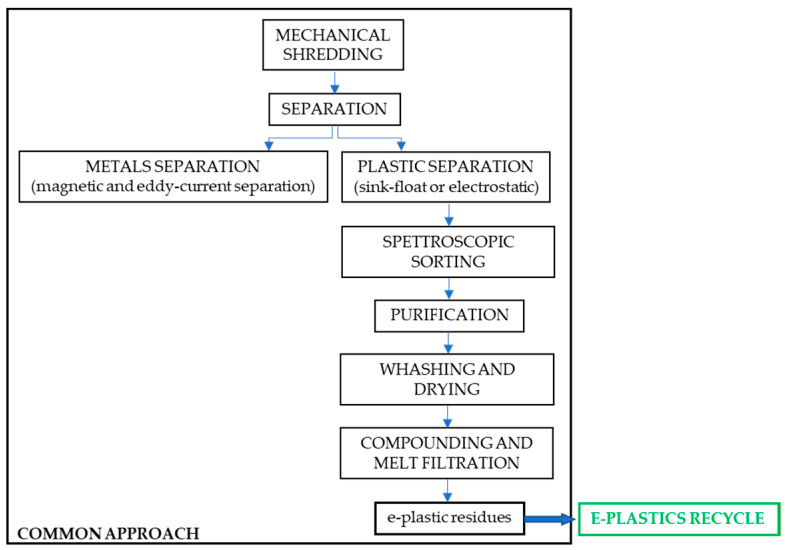
Mechanical recycling (common approach).

After recovering the precious metals, reprocessing the recoverable plastics for recycling purposes involves feedstock (chemical) recycling (to convert e-plastics into fuel, monomers, or other chemicals) or mechanical recycling. Mechanical recycling (Figure 1) refers to primary recycling, with WEEEP reprocessing to obtain new plastic products, but also to down-cycling, with the re-use of plastic materials for other purposes.

The “standard” mechanical approach can be effectively applied to the most common e-plastics, such as ABS, HIPS, and PP, which are available in large volumes. When a large mix of materials has to be reprocessed, dismantling-based recycling (i.e., the disassembly of plastic components before shredding) is the most effective method for recovering high-quality post-consumer plastics (primary recycling) [29]. Using optimized strategies allows for the separation of plastics so that different materials can be recycled individually [1,30] and the presence of hazardous substances can be measured [31,32] for use as secondary raw for chemical recycling [27].

All the recycling technologies have a specific impact on the environment and on the human health of workers and the general population, depending also on geo-spatial characteristics, socio-political aspects, and a regulatory framework [26]. In most LMICs, the informal recovery of materials from WEEE does not use optimized methodologies, but it takes place in small workshops, using different techniques and rudimentary equipment. The metals are mainly recovered by heating e-waste over an open flame or a hot plate, and the remaining non-reusable components are ground mechanically to further recover metals; in some cases, e-waste is mechanically shredded to recover metals, and plastic is ground for recycling or dumped without any control. These unregulated procedures expose workers, the general population, and the environment to hazardous contaminants [33].

The effectiveness of tailored dismantling routes was underlined by Stevels et al. [34], who analyzed the advantages of a deep disassembly strategy in LMICs, which was deemed feasible because of low labor costs. Deep disassembly limits investment requests in equipment for mechanical treatment, and entails the obtainment of purer WEEE fractions, with better yields and lower upgrading costs. Moreover, it improves eco-efficiency, reduces waste, and contributes to more general sustainability goals, including the health and safety of local communities.

#### 2.1.2. BFRs in E-Plastics

The BFRs are the most effective flame-retardant agents and among the most investigated hazardous e-plastic additives [7]. The main group of BFRs includes brominated flame retardants with antimony (Sb) as a synergist (e.g., polybrominated diphenyl ethers (PBDEs), decabromodiphenylethane, and tetrabromobisphenol A (TBBPA)). The typical BRF concentrations in e-waste plastics range between 3 and 25% *w/w* [35].

The most employed BFR is TBBPA (and its derivatives), which is used as a reactive additive polymerized to epoxy and polycarbonate resins (PC) or is added to acrylonitrile-butadiene-styrene (ABS), polystyrene (PS), high-impact polystyrene (HIPS), and thermoplastic polyesters (PET/PBT). It contains 58.4% bromine and emits bromine vapors when heated to decomposition (at 200–300 °C) [36]. It is lipophilic, with a moderately high octanol/water partition (log K_ow_ = 5.9), and some studies have shown that it can bioaccumulate with repeated exposure [37]. It can be found both in large and small household appliances, including TVs, and telecommunication equipment [38]. Though more recent electronic devices contain BFRs and TBBPA well below the limit values imposed by current regulations, older models that are still significantly present within the e-waste streams at a global level contain higher amounts of hazardous substances. Recent measurements performed by Singh et al. [39] on cellular phones and smartphones and by Jandric et al. [40] on six WEEE devices (IT and small appliances) confirmed this trend.

TBBPA and/or its derivatives might leach from these products into the environment: TBBPA is susceptible to photolysis, and reductive de-bromination has been observed under experimental conditions. When the POP concentration is still higher than EU regulatory limits, the authorized processing is feedstock recycling, incineration, or energy conversion in controlled conditions, avoiding inappropriate end-of-life management processes. Primary recycling of e-plastics in the presence of BFRs can cause the unintentional contamination of children’s toys [41], polymeric kitchenware, food packaging, and food containers [42], probably due to the global e-waste streams and under-controlled recycling [35].

Delva et al. [43] found that a few studies examined the actual reprocessing of WEEP and focused on the influence of FRs. Analysis to detect the presence of BFRs in e-plastics (X-ray fluorescence spectrometry or near-infrared) and sorting techniques help in recycling “bromine-free” plastics; the recycling rate of flame-retarded plastics through sink float separation is up to 70%, while non-flame-retarded plastics have a recycling rate of 65% when separated by the electrostatic method [44]. Some advanced processes (Creasolv^®^ and Centrevap^®^) have been developed for removing BFRs from WEEE. In a safe work environment, upgrading strategies can be applied to mechanical recycling using brominated waste streams instead of virgin polymers in applications requiring flammability; the recycling of e-plastic containing TBBPA can maintain its original properties and fire grade during four extrusions.

#### 2.1.3. Flame Retarded E-Plastic Toxicity

Many authors have demonstrated the high human health risks and environment detrimental impacts of improper handling, processing, and disposal of WEEE, mainly in developing countries. Bakhiyi et al. [20] analyzed the scientific literature on the theme, presenting a comprehensive and critical overview of the toxic potential of WEEE.

Within unregulated WEEE recycling, workers without adequate means and training operating on unsafe sites are exposed to toxic contaminants; persistent and bioaccumulative organic pollutants, including BFR, are discharged into the environment, affecting the air, surface water, groundwater, soil, sediment, food, and wastewater. TBBPA has been detected in food packaging, sludge, sediments, and biota samples [38]. Human exposure to TBBPA can occur through inhalation, ingestion, or dermal contact with devices containing it [45]; Hagmar et al. (2000) [46] evaluated a TBPPA half-life of 2.2 days for exposed workers. Usenko et al. [47] compared the toxicity of nine BFRs using the Zebrafish embryo model and concluded that exposure to TBBPA may pose a risk to human health greater than that of PBDEs. In addition, some recent research emphasizes the need for deeper investigations on novel BRFs, in particular on TBBPA derivative toxicity, since preliminary research indicates that some of them can cause cancer and genetic effects [48]. In Table 1, the main types of toxicity related to e-plastics containing flame retardants are listed.

In addition, associated with WEEE processing, secondary contaminants are released. In Table 2, the main type of toxicity related to the processing of e-plastics containing flame retardants is shown.

#### 2.1.4. Re-Use of E-Plastics as Construction Material

There is general agreement among researchers concerning the need for tailored solutions for e-waste recycling, based on local recycling practices and economies, in order to protect the health of workers and their families and preserve the environment [49]. One relevant research effort is dedicated to the potential applications of e-waste in construction, such as cathode ray tubes; the incorporation of e-plastics in green concrete or in bituminous mixes are considered viable options for low-income economies [21].

Santhanam et al. [50] investigated the use of a share of e-plastic powder in bituminous-grade VG30 used in pavement (with applications in India) as an alternative to conventional bitumen. The e-plastics were collected from PC boards, phones, and other electronic appliances, discarding fractions containing metals, such as lead, lithium, copper, and aluminum. The test results showed that 10% of e-plastic powder can be used in pavements along with the conventional bitumen for better strength. Makri et al. [51] recovered ABS from plastic houses of liquid crystal display (LCD) screens for use as RPAs in cement mortar and obtained samples with appropriate physical properties. Kumar et al. [52] showed the feasibility of using e-plastic (HIPS) as a partial replacement of coarse aggregates in concrete.

Gomez et al. [53] developed a stabilization process through a core-shell approach where the e-plastic particles were covered with a mixture of cement and additives. Starting from a mix of e-plastics supplied, after grinding, by a recycling company processing all types of WEEE, they produced the core-shell RPAs and characterized the samples mechanically and chemically. The leaching of TBBPA from naked e-plastic particles and core-shell RPAs was detected by means of gas chromatography analysis. The novel material shows good physical properties and an effective stabilization of TBBPA, where leaching is avoided.

#### 2.1.5. E-Plastic Toxicity Assessment

In the last few years, many LCA studies related to WEEE management strategies and systems have been performed [54,55], while fewer studies that specifically address the recycling of plastic fractions originating from WEEE treatment have been conducted.

Bientinesi and Petarca [56] compared the environmental impact of two thermal treatment systems designed for plastic from WEEE: the combustion in an MSW plant in Germany and the gasification in a gas turbine system in the Netherlands. Wäger and Hischier [57] investigated the environmental life cycle impacts associated with the production of post-consumer plastics from WEEE treatment residues in a plastic recycler plant located in Austria and stated that the recycling of e-plastic residues is superior to alternative disposal. Jonkers et al. [58] performed an LCA study to compare the environmental impacts of BFRs and halogen-free flame retardants (HFFRs) in an electronic product over the whole life cycle and demonstrated that improper treatment of WEEE has the highest impact compared with different waste treatment options. Jaunich et al. [59], focusing on CO_2_ emissions released in all steps of the recovery and recycling processes, developed a holistic framework to estimate the cost and environmental impacts associated with e-waste management.

LCA has been combined with material flow analysis by several authors. De Meester et al. [60] optimized the WEEE recycling chain in Belgium. Fiore et al. [10] analyzed WEEE management in an Italian full-scale plant. Wäger et al. [61] calculated the overall environmental impacts of collection, pre-processing, and end-processing of Swiss WEEE collection and recovery systems.

With regard to LMICs, previous studies have addressed both environmental and social aspects. Pathak et al. [62] compared the CO_2_ emission reduction of a conventional e-waste recycling process with that of a greener treatment in India and demonstrated the positive effects deriving from the proper handling and treatment of e-waste. Aparcana and Salhofer [63] developed a methodological procedure for assessing the contribution of formalized recycling systems, in comparison with informal systems, in low-income countries in terms of social impacts.

### 2.2. LCA Methodology

#### 2.2.1. Goal and Scope

The goal of this study was to answer the second research question that addresses the environmental and human risks related to WEEE and e-plastic management in developing countries.

#### 2.2.2. Functional Unit and System Boundaries

Plastic deriving from electronic waste after collection is defined as a functional unit. An amount of 1 kg is considered as reference flow, and all the results are associated with this quantity [64]. The system boundaries include the informal treatment process steps and different end-of-life scenarios derived from the literature review, including common disposal practices, such as incineration and landfill, and different re-use solutions for e-plastic scrap in construction materials. All the transports involved in the life cycle are excluded from the study, as the focus is the end-of-life phase of e-plastic residues after being treated.

**Figure 2:**
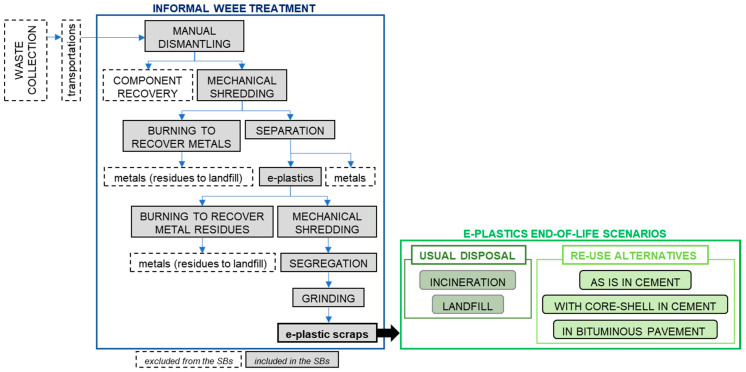
System boundaries for the informal treatment and the end-of-life scenarios for e-plastics.

The processes analyzed are shown in Figure 2 and are described below.

#### 2.2.3. Inventory Analysis

**Figure 3:**
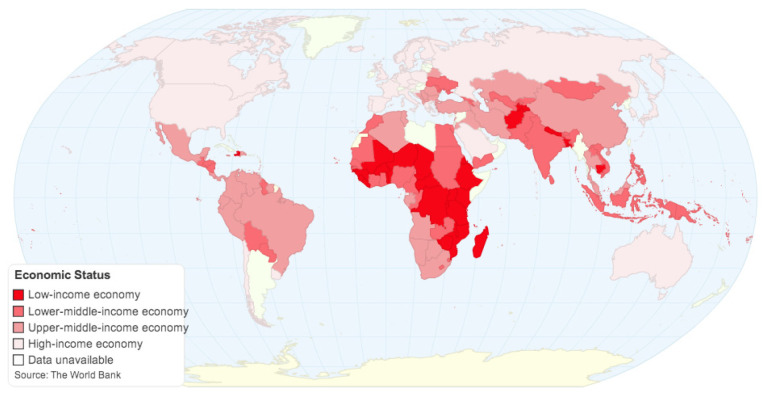
Map of Developing and Developed Countries (as determined by GNI per capita) elaborated according to the World Bank (2016), ChartsBin.com, viewed 25 September 2020, <http://chartsbin.com/view/25857>.

In this study, inventory data were obtained from different sources, such as scientific literature, reports, and the Ecoinvent 3.0 LCA-database [65]. Data on the quantity, e-waste composition, and recycling rate were obtained from studies related to WEEE management in China, India, and Zambia [18,62,66,67], assumed to be representative of the situation in developing countries (Figure 3).

#### 2.2.4. Informal Treatment

The informal treatment process conventionally starts with a manual dismantling activity that leads to the recovery of valuable materials and components that can be re-used. The remaining material is mechanically shredded into components that can be separated to recover metals. A fraction is burned in an open site to recover metal scraps. In this study, the residues of the incineration processes are supposed to be landfilled. The remaining fraction, after segregation and grinding, constitutes a residue composed by plastic containing toxic residues, such as flame retardants. This material can be subjected to different end-of-life treatments, such as incineration, landfill, and re-use in construction materials, analyzed, and compared in the present LCA study.

The modelled processes were based on Ecoinvent 3.0 datasets. The specific data and assumptions used for the modelling are listed in Table S1. The waste input materials and elements, such as the Cu, Sb, Bi, Cd, and Ag in air and the Cu, Fe, Ni, Pb, and Zn in water and soil, were adjusted according to the literature data [20,53,62,68].

#### 2.2.5. End-of-Life Alternative Scenarios

The five considered alternative end-of-life scenarios are described in Table 3, while the data and assumptions used for the end-of-life scenarios life cycle modelling are presented in Table S2.

##### Incineration Scenario (WEEP-I)

The model of this scenario is based on the process sheet, ‘Waste plastic, consumer electronics {GLO}| treatment of waste plastic, consumer electronics, open burning | APOS, U’, representing informal or uncontrolled burning, based on municipal waste incineration without any pollution control and suggested by Ecoinvent 3.0 as suitable for informal recycling (e.g., burning of e-waste) [53].

##### Landfill Scenario (WEEP-L)

The other most common practice in developing countries is WEEE disposal, often with household waste, leading to toxic emission and hazardous leachate [62]. The model of this scenario is based on the Ecoinvent 3.0 process sheet ‘Waste plastic, consumer electronics {GLO}| treatment of waste plastic, consumer electronics, unsanitary landfill, wet infiltration class (500 mm) | APOS, U’, representing the unsanitary sub-controlled landfill for municipal solid waste [65].

##### Re-Use in Cement Scenario (WEEEP-RC and WEEEP-RCS)

Foreground data on the re-use of e-plastics in cement were obtained by Gómez et al. (2020), who proposed a methodology to encapsulate WEEEP with cement in order to obtain a safe and optimal material for use in civil construction. They compared the mechanical and chemical properties of cement with as-is e-plastic (WEEEP-RC) and cement with e-plastics encapsulated in a core-shell (WEEEP-RCS) that is able to limit releases of tetrabromobisphenol-A (TBBPA) and styrene derivatives. The cement mix composition and the relative releases considered are listed in Table 4.

##### Re-Use as is in Bituminous Pavement Scenario (WEEEP-RBP)

Inventory data on the re-use of plastics in bituminous pavement were obtained by Santhanam et al. [50], who added e-waste plastic powder as an alternative to conventional bitumen in a layer of flexible pavement. The 10% bitumen replacement with e-waste plastic powder was considered in the study because it showed better strength.

#### 2.2.6. Impact Assessment

Recommended as one of the best models for LCIA on toxicity [69], the USEtox (recommended+interim) V2.02 method [70,71] was utilized to assess the environmental profile of informal WEEE treatment and of e-plastic end-of-life scenarios in order to address the human toxicological and ecotoxicological impacts [72]. The following impact categories were considered: “human toxicity, cancer”, “human toxicity, non-cancer”, and “freshwater ecotoxicity”. In addition to the aggregated total, the two damage categories “human health” and “ecosystems” are shown and discussed.

For human toxicity, the USEtox model calculates characterization factors for carcinogenic impacts, non-carcinogenic impacts, and total impacts (carcinogenic and non-carcinogenic aggregated, assuming equal weighting at the midpoint level or impact-specific weighting at the end-point level, where, in the latter, carcinogenic and non-carcinogenic impacts are weighted differently) of substance emissions on household indoor air, occupational indoor air, urban air, continental rural air, continental freshwater, continental (coastal) sea water, continental agricultural soil, and/or continental natural soil. Human toxicity potentials at the midpoint level are expressed as comparative toxic units (CTUs) per kg emission.

The unit of the characterization factor for freshwater ecosystem toxicity is a potentially affected fraction of species (PAF) at the mid-point level and a potentially disappeared fraction of species (PDF) at the endpoint level, integrated over the freshwater volume (m^3^) and the duration of 1 day (d) per kg emission. The unit of USEtox characterization factors for human toxicity is disability-adjusted life years (DALYs) [73,74].

The USEtox impact assessment categories are summarized in Table 5.

## 3. Results

### 3.1. LCIA Results Associated with Informal E-Plastic Treatment

**Figure 4:**
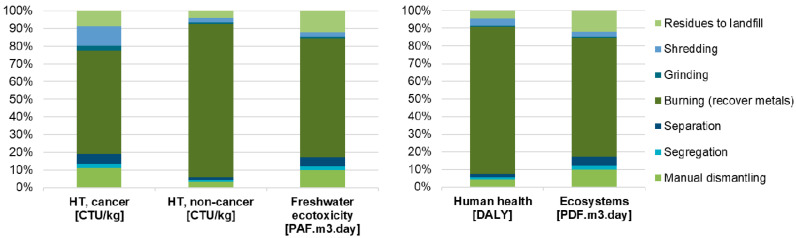
Environmental impacts associated with the informal treatment of WEEE, with evidence of the impacts related to the individual process steps for 1 kg of e-plastics (left: USEtox mid-point results for the “human toxicity, cancer”, “human toxicity, non-cancer”, and “freshwater ecotoxicity” impact categories; right: USEtox end-point results for the “human health” and “ecosystems” damage categories.

Figure 4 shows the results of the various mid- (left side) and end-point (right side) indicators from USEtox for the informal treatment of 1 kg of e-plastics, with evidence of the impacts related to the individual process steps. The networks of the USEtox mid- and end-point results are shown in Figures S1 and S2.

The burning of e-waste to recover metals contributes to the overall impact in all categories. In the case of “human toxicity, cancer”, the burning process accounts for about 59%, mainly due to nickel and dioxins emissions into the air and mercury emissions into the soil. Other prevalent impacts are caused by the shredding process, which accounts for about 14%, manual dismantling (10%), and the landfill of residues (9%). In the case of “human toxicity, non-cancer”, the high impact is caused by mercury emissions to air; antimony, cadmium, and arsenic emissions to soil; and cadmium emission into groundwater. The “freshwater ecotoxicity” is mainly affected by copper emissions into the soil and groundwater.

In both end-point categories, the impact of the informal treatment of WEEE is largely dominated by the burning process, which accounts for about 83% of the “human health” impact and about 67% of the “ecosystems” impact. In the first case, the higher impact is mainly due to the direct emissions of mercury; in the second case, it is mainly due to copper emissions into the soil and groundwater.

### 3.2. LCIA Results Associated with End-of-Life Alternative Scenarios

**Figure 5:**
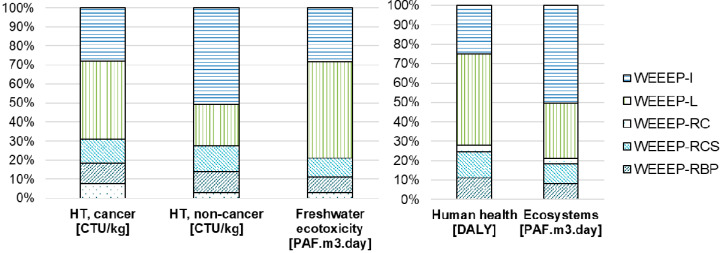
Environmental impacts associated with the five end-of-life scenarios (WEEEP-I, WEEEP-L, WEEEP-RD, WEEEP-RCS, and WEEEP-RBP) for 1 kg of e-plastic residues (left: USEtox mid-point results for the “human toxicity, cancer”, “human toxicity, non-cancer”, and “freshwater ecotoxicity” impact categories; right: USEtox end-point results for the “human health” and “ecosystems” damage categories.

The comparison between the five end-of-life scenarios (WEEEP-I, WEEEP-L, WEEEP-RD, WEEEP-RCS, and WEEEP-RBP) for 1 kg of e-plastic residues is shown in Figure 5, with evidence of the impacts affecting each category due to the single option. The left side of the figure shows the values relative to the mid-point categories, while the right part addresses the end-point categories. The detailed results are presented in Table S3.

With the exception of “human toxicity, non-cancer”, the landfill scenario (WEEEP-L) dominates among impacts at the mid-point level, accounting for almost 40% of the “human toxicity, cancer” impact and about 50% of the “freshwater ecotoxicity” impact, which are both affected mainly by nickel and copper releases into groundwater. The treatment steps account for almost 25%. In the case of the “human toxicity, non-cancer” impact, the incineration (WEEEP-I) has the highest impact, contributing almost 50%, caused mainly by mercury emissions to air and antimony releases to soil. The processes related to re-use as is in cement (WEEEP-RC) contribute about 15% of the “human toxicity, cancer” impact, 20% of the “human toxicity, non-cancer” impact, and 10% of the “freshwater ecotoxicity” impact due to the release of substances, such as nickel to soil, mercury to air, and copper to soil and groundwater. The re-use of aggregates in cement from WEEE plastic though a core-shell strategy (WEEEP-RCS) allows for a reduction in environmental impact of almost 6% in all mid-point impact categories. The re-use of e-plastic residues in bituminous pavement is responsible for about 9% of the “human toxicity, cancer” impact and for less than 5% in the other two mid-point categories. Observing the end-point results, the “human-health” damage category is dominated by processes related to the WEEEP-L scenario, while “ecosystems” are mainly affected by the WEEEP-I scenario. The WEEEP-RPB scenario has the lowest impact in both damage categories (less than 5%), followed by the two scenarios related to the re-use in cement. For all categories and the three recycling options, the treatment phase accounts for about 10%.

### 3.3. Results Interpretation

The results of the treatment phase confirm that unsafe electronic waste handling and improper techniques to recover components release hazardous substances to the environment with consequent negative effects on humans and ecosystems. In particular, the burning of e-plastics in open air to recover metals and the landfilling of residues in open dumps was found to predominantly affect all the considered impact categories due to mercury and copper releases.

The overall results demonstrate that the environmental impact values for the disposal phase depend very much on the scenario [75]. Not surprisingly, the re-use of e-plastic scraps has been found to be beneficial in lowering environmental burdens, compared to the landfill or incineration scenario.

A significant reduction of environmental impact was found in the WEEEP-RPB scenario, which was 90% lower than the usual landfill and incineration scenarios and 68% lower than the re-use in cement scenarios. These latter scenarios are mainly affected by the potential releases of TBBPA but also by the use of cement, demonstrating that these disposal methods also carry heavy environmental burdens, depending on the materials [76].

### 3.4. Sensitivity Analysis

In order to evaluate the relevance and influence of inventory data, several sensitivity analyses were performed by varying data directly related to the e-plastic treatment and alternative end-of-life scenarios. In particular, the sensitivity analysis addresses the emissions into the air, soil, and water. Regarding the incineration and landfill treatment options, the most impact-relevant heavy and toxic substances were reduced by 10%, assuming more optimistic scenarios. For both scenarios involving the re-use of cement, TBBPA leachate was modified, according to the experimental results shown by Gómez et al. [53]. For the last scenario, namely the re-use as is in bituminous pavement, substances, such as aluminum, zinc, chlorides, and sulphates, were included, according to Jullien et al. [77], who performed an LCA for the leaching of granular alternative materials used in road construction, including waste, assuming a similar soil and water leachate. Table 6 gives an overview of the considered sensitivity options relative to the corresponding steps of each process.

**Figure 6:**
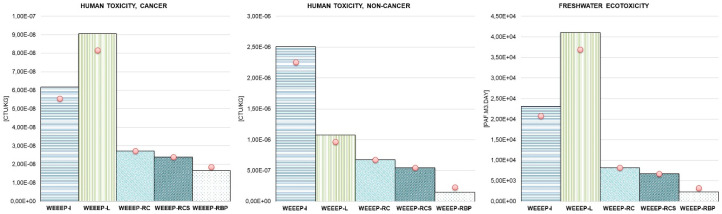
Environmental impacts associated with the five end-of-life scenarios (WEEEP-I, WEEEP-L, WEEEP-RD, WEEEP-RCS, and WEEEP-RBP) for 1 kg of e-plastic treatment residues including the influence of the sensitivity options (S1). Shown are the USEtox mid-point results for the impact categories “human toxicity, cancer”, “human toxicity, non-cancer”, and “freshwater ecotoxicity”.

**Figure 7:**
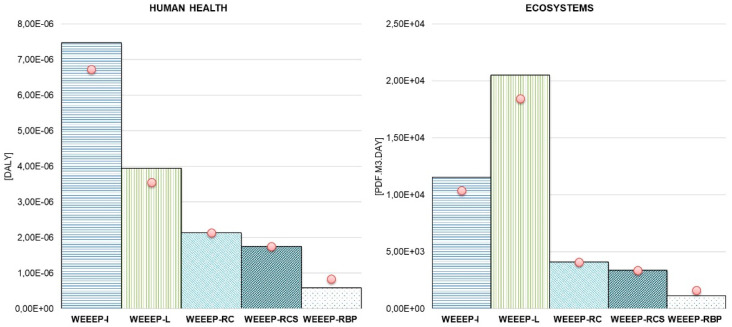
Environmental impacts associated with the five end-of-life scenarios (WEEEP-I, WEEEP-L, WEEEP-RD, WEEEP-RCS, and WEEEP-RBP) for 1 kg of e-plastic treatment residues including the influence of the sensitivity options (S1). Shown are the USEtox end-point results for the damage categories “human health” and “ecosystems”.

The influence of all these sensitivity analyses on the investigated end-of-life e-plastic scenarios is shown in Figure 6 for the three impact categories and in Figure 7 for the two damage categories of the USEtox method. The bars indicate the results for the default processes, while the red circles highlight the results for the sensitivity analysis of each alternative.

The results related to the re-use in cement as is or with cement shells clearly show that the sensitivity analysis of the input emissions has no relevant influence on the overall categories. A heavy and toxic emission reduction of almost 10% would decrease the impacts of landfill and incineration processes by 10% to 30%, but they would still remain the most impactful end-of-life scenarios. The consideration of potential leachate to soil and water in the case of the re-use of e-plastics in bituminous pavement would increase the environmental impact by almost 10% in all categories.

## 4. Discussion

As the literature review shows, besides the global concern for the environmental and human health risks caused by the unregulated treatment practices of e-plastics containing BFRs, some tailored end-of-life options could reduce the impact of these materials with respect to uncontrolled incineration or landfilling. The most analyzed uses of e-plastics are in cement and in bituminous mix; a diverse mix of plastics and options have been analyzed, with a focus on the technical and structural feasibility. Interestingly, Gomez et al. [53] emphasized the hazardous potential of re-using flame-retarded e-plastics as RPAs in cement mortar, suggesting a simple stabilization procedure to avoid TBBPA leaching.

In order to support the techno-economic considerations, this study applied the LCA methodology to explore informal WEEE treatment and some of the most analyzed e-plastic end-of-life scenarios, addressing human health and environmentally toxic impacts in the context of LMICs, aiming to cover a gap in the literature. To the best of our knowledge, indeed, LCA studies of WEEE management and of the recycling of e-plastics in developing countries are not available in the literature. Moreover, most of the studies on environmental impacts deriving from plastics originating from WEEE do not consider additives, such as FR [58]; moreover, the additive content is ignored in the studies on the environmental impacts of e-waste treatments and on the re-use of e-waste, although additives constitute around 10% of the weight of plastics and can account for 5–40% of their cradle-to-grave greenhouse gas (GHG) emissions. Considering both environmental and health concerns is inevitable when evaluating e-waste treatments and the re-use of e-plastics, especially in developing countries where informal e-waste treatments and recycling methods are used. In fact, the end-of-life phase could have lower negative impacts in terms of CO_2_ emissions but higher negative impacts in terms of ecotoxicity, human exposure, and health concerns [78].

The applied USEtox model allows for an evaluation of the ecotoxicity risks deriving from the treatment and recycling of the plastic fraction of electric and electronic waste at informal workshops, and a comparison of chosen end-of-life scenarios. Answering research question 1, in addition to the incineration and landfilling scenarios, the considered e-plastic reuse options, suitable for applications in low-income economies, are the use in bituminous pavement and as RPAs in cement, considering both the reuse as is and the stabilization procedure in core shells.

The outcomes for research question 2 show that the burning of WEEE in an open site to recover metals is one of the most impactful steps in informal treatment, mainly due to nickel and dioxin emissions into the air and mercury emissions into the soil, as expected from direct measurements taken at some informal treatment sites [18,62,66,68]. The cited emissions have a negative effect on all the selected categories; in particular, they account for almost 92% of the “human toxicity, non-cancer” and “human health” impacts. Our results draw attention to the whole treatment chain, showing that processes, such as unsafe handling and dismantling or uncontrolled shredding in informal processing structures, without personal protective equipment or filtration systems, have an impact on human health and ecosystems that cannot be neglected when evaluating the e-plastic management. This finding supports quantitatively previous warnings in the literature, according to which the improper handling and management of e-waste are some of the main causes of environmental pollution and degradation in several cities, particularly in developing countries, because of a lack of regulations and appropriate treatment facilities [18].

The analysis of the comparison between alternative end-of-life scenarios shows that the recycling and re-use of e-plastics inside construction materials are environmentally superior to alternative strategies, such as incineration and disposal, and these superior strategies have an environmental impact that accounts for less than 50% of the impact in the mid- and end-point categories considered in this study. These results are consistent with previous studies on WEEEP treatment residue management in European countries, such as Austria [57]. Sensitivity analyses show that variations in the assumed parameters do not change the overall conclusion.

Between the three recycling alternatives, the re-use of e-plastics in bituminous pavement has the lowest environmental impact with respect to re-use in cement components. It should be noted that the standard scenario does not consider potential leaching and percolate. The sensitivity analysis, in fact, including emission literature data, has a direct influence on the total environmental impact of the scenario, reducing the gap with re-use in cement scenario by about 10%.

Between the two scenarios that consider the recycling of e-plastics in cement matrixes, the one where plastic components are encapsulated in a cement matrix shows a 10% lower environmental impact thanks to the reduction of TBBPA and formaldehyde releases.

However, the cited findings might not be generalizable, considering that the present work clearly has some limitations. First of all, the inventory data are based on literature and assumptions and do not exactly correspond to a site-specific scenario. Secondly, the study only investigated some of the re-use alternatives that are available in the literature and are commonly practiced in LMICs. Moreover, the impact assessment focused only on potential ecotoxicity risks, not on other environmental indicators, such as climate change or water consumption.

Although affected by some limitations, the results of the present research show that these quite simple technologies, suitable for implementation in small factories and requiring low investment, are an advisable option in LMICs, since they reduce ecotoxicity risks with respect to the current disposal strategies. Further research will have to consider both environmental aspects and aspects related to the investigation of potentially hazardous emissions and leachate released by e-plastics during their re-use in construction materials over mid- to long-term periods and under different conditions; additional important phases, such as maintenance, should also be considered [79].

Moreover, alternative solutions to avoid the environmental risks associated with FRs to FRs in plastic should be investigated in an eco-design approach. The use of FRs with a low environmental load is an effective solution [45], keeping in mind that FRs cannot be simply interchanged, as most FRs are compatible with only a limited amount of polymers [58]. However, since the end-of-life phase has the largest environmental load, future improvements are expected, such as investigations of alternative recycling and re-use possibilities and studies on disassembly (and consequently assembly) techniques [30] that lead to decreases in the amount of e-plastic scraps [80] and allow for effective recycling and remanufacturing strategies.

## Figures and Tables

**Table 1:** Toxicity related to e-plastics containing flame retardants [20].

Contaminants	Main Type of Toxicity
Halogenated flame retardants:	
Brominated flame retardants (e.g., PBDEs, TBBPA, and HBCD)	Endocrine disrupter and neurotoxic
Novel brominated flame retardants (e.g., DBDPE, TBPH, and TBBPA-BGE)	
Chlorinated flame retardants	An endocrine disrupter, neurotoxic, and carcinogenic
Non-halogenated flame retardants:	
Organophosphorus-based flame retardants	An endocrine disrupter
Nitrogen-based flame retardants	Nephrotoxic and neurotoxic
Antimony ^1^	Lung, eye, and gastro-intestinal irritant

^1^ Often associated with halogenated and non-halogenated flame retardants [31]. Legend: DBDPE, decabromodiphenyl ethane or 1,2-bis(pentabromodiphenyl)ethane; TBBPA, tetrabromobisphenol A; TBBPA-BGE, tetrabromobisphenol A-bis bis(glycidyl)ether; TBPH, bis(2-ethylhexyl)-3,4,5,6-tetrabromo- phthalate.

**Table 2:** Toxicity related to e-plastics containing flame-retardant processing [20].

WEEE Processing	Contaminants	Contaminants’ Source	Main Type of Toxicity
Combustion (PCBs, plastics, PVC)	PAHs	Brominated, chlorinated, and organophosphorus FRs in plastics and from PVC	Carcinogenic and a photosensitizer
Incineration of e-waste residues as a disposal strategy	PCDD/Fs PBDD/Fs PXDD/Fs		Immunotoxic, carcinogenic, reprotoxic, endocrine disrupters, may induce birth defects and dermal damage (chloracne)
	Bisphenol A	From the combustion of polycarbonate plastic	An endocrine disrupter
	Acids	E.g. hydrobromic acid from brominated FRs, hydrochloric acid from chlorinated FRs and from incomplete combustion of PVC, and phosphoric acid from organophosphorus FRs)	Induces mild to severe burns to the eyes and skin, sore throat, respiratory problems, and corrosive injuries to lips, mouth, throat, etc., if swallowed

Legend: PBDD/Fs, polybrominated dibenzo-p-dioxins and dibenzofurans; PAHs, polycyclic aromatic hydrocarbons; PCBs, polychlorinated biphenyls; PCDD/Fs, polychlorinated dibenzo-p-dioxins and dibenzofurans; PVC, polyvinyl chloride; PXDD/Fs, mixed polybromochloro-dibenzo-p-dioxins and dibenzofurans.

**Table 3:** Built end-of-life scenarios.

End-of-Life Scenarios	Description
WEEP-I	Open burning
WEEP-L	Open landfill
WEEEP-RC	Re-use in cement as is
WEEEP-RCS	Re-use in cement with a core shell
WEEEP-RBP	Re-use as is in bituminous pavement

**Table 4:** Inventory data for e-plastic re-use in cement alternatives. Adaptation from Gómez et al. [53].

Re-Use in Cement	Aggregate	Quantity [g]	OPC [g]	Water [mL]	TBBPA Leachate ^2^	Styrene Derivatives
As is (WEEEP-RC)	WP	131	50	26	(23.0 ± 0.1)	1
With core-shell (WEEEP-RCS)	WP@OPC:PPR:AC ^1^	154	50	25	(19.4 ± 0.8)	- ^3^

^1^ WP@OPC:PPR:AC was made using 500 g of WP (waste plastic), 300 g of OPC (ordinary CPF40 Portland cement), 300 g of PPR (polish porcelain residue) dried in oven at 110 °C for 24 h, 50 g of AC (powder activated charcoal) used as received, and 240 mL of water. ^2^ TBBPA leachate from samples in mgTBBPA/kgWEEEP (×101 mg/kg). ^3^ Not available data.

**Table 5:** USEtox impact assessment categories.

Impact Assessment Categories	u.m.
mid-point level	
HT, cancer	CTU/kg
HT, non-cancer	CTU/kg
freshwater ecotoxicity	PAF.m3.day
end-point level	
human health	DALY/kg
ecosystems	PDF.m3.day

Legend: HT, human toxicity; CTU, comparative toxic units; PAF, potentially affected fraction of species; DALY, disability-adjusted life years; PDF, potentially disappeared fraction of species.

**Table 6:** Parameters used for the sensitivity analysis.

End-of-Life Scenarios	Sensitivity Parameters
WEEEP-I	Heavy and toxic air and soil emissions (PAH, PCDD, PBDD, PXDD, and Bisphenol A) (Bakhiyi et al., 2018; Awasthi et al., 2019) I (S1): −10% default
WEEEP-L	Heavy and toxic soil and water contaminants (Cu, Fe, Pb, and Zn) (Pathak et al., 2017) L (S1): −10% default
WEEEP-RC	TBBPA leachate RC (S1): (4.4 ± 0.1) TBBPA leachate (Gómez et al. (2020))
WEEEP-RCS	TBBPA leachate RCS (S1): <LOQ ^1^ TBBPA leachate (Gómez et al. (2020))
WEEEP-RBP	Heavy and toxic soil and water emissions RBP (S1): added according to Jullien et al. (2019)

^1^ Limit Of Quantification (LOQ = 281 mg/L).

## Supplementary Materials

The following are available online at https://www.mdpi.com/2305-6304/8/4/99/s1, Figure S1: Network of the USEtox end-point results for the damage category “Human Health”, Figure S2: Network of the USEtox end-point results for the damage category “Ecosystems”, Table S1: Data and assumptions used for the informal treatment life cycle modelling, Table S2: Data and assumptions used for the end-of-life scenarios life cycle modelling, Table S3: Environmental impacts associated with the five end-of-life scenarios.
