# Supplementary material for: Ecotoxicity of Plastics from Informal Waste Electric and Electronic Treatment and Recycling
Source: Toxics. 2020 Nov 8;8(4):99. doi: 10.3390/toxics8040099 (PMC7712128; doi:10.3390/toxics8040099)
Supplement: Supplementary file 1 [file toxics-08-00099-s001.pdf]

Supplementary Materials: Ecotoxicity of Plastics from Informal Waste Electric and Electronic Treatment and Recycling

Maria Angela Butturi, Simona Marinelli, Rita Gamberini and Bianca Rimini

Table S1. Data and assumptions used for the informal treatment life cycle modelling.

Process Step	Description of Main Data and Assumptions
Weeep	Input data and assumptions according to [1,2]. It is assumed a production volume of 3310 kg/year and a polymer distribution of e-plastics of 35% of acrylonitrile butadiene styrene (ABS), 25% of high impact polystyrene (HIPS), 30% polypropylene (PP) and 10% of polycarbonate (PC).
Manual dismantling, segregation and separation	Input data and assumptions according to [1]. Dataset "Waste plastic, consumer electronics {GLO}∣ treatment of waste plastic, consumer electronics, unsanitary landfill, wet infiltration class (500mm) ∣ APOS, U " from Ecoinvent 3.0 is used.
Burning	Input data and assumptions according to [1,2,3]. It is assumed that the 30% of e-plastics is burned to recover metals and metals residues. Dataset "Waste plastic, consumer electronics {GLO}∣ treatment of waste plastic, consumer electronics, open burning ∣ APOS, U" from Ecoinvent 3.0 is used.
Sgrinding and shredding	Input data and assumptions according to [1,2]. Dataset " Shredding, electrical and electronic scrap/GLO U" from Ecoinvent 3.0 is used.
Landfill of residues	Dataset "Waste plastic, consumer electronics {GLO}∣ treatment of waste plastic, consumer electronics, unsanitary landfill, wet infiltration class (500mm) ∣ APOS, U" from Ecoinvent 3.0 is used.

Table S2. Data and assumptions used for the end-of-life scenarios life cycle modelling.

Process Step and Scenario	Description of Main Data and Assumptions
Incineration	Input data and assumptions according to [2]. Dataset 'Waste plastic, consumer electronics {GLO}∣ treatment of waste plastic, consumer electronics, open burning ∣ APOS, U' from Ecoinvent 3.0 is used.
Landfill	Input data and assumptions according to [2]. Dataset 'Waste plastic, consumer electronics {GLO}∣ treatment of waste plastic, consumer electronics, unsanitary landfill, wet infiltration class (500mm) ∣ APOS, U' from Ecoinvent 3.0 is used.
Weep-rc and weep-rcs	Input data and assumptions according to [4]. Datasets 'Waste plastic, consumer electronics {GLO}∣ treatment of waste plastic, consumer electronics, unsanitary landfill, wet infiltration class (500mm) ∣ APOS, U'; "Cement, Portland {RoW}∣ market for ∣ APOS, U" from Ecoinvent 3.0 are used. It is assumed that "2,2-Bis(4-hydroxy-3,5-dibromophenyl)propane" and "styrene" emissions are released to air, water and soil.
Weep-rbp	Input data and assumptions according to[5,6]. Dataset "Bitumen adhesive compound, hot {GLO}∣ market for ∣ APOS, U" from Ecoinvent 3.0 is used. It is assumed that Al, Cd, Cr, Cu, Fe, Mn and other emissions are released to water and soil.

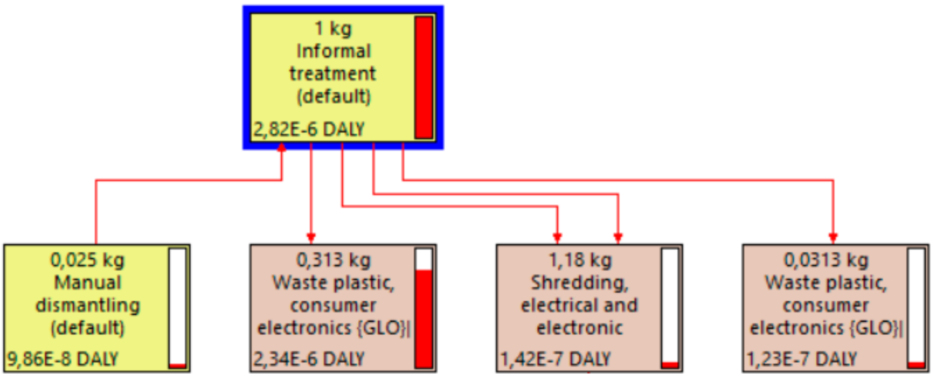

Figure S1. Network of the USEtox end-point results for the damage category "Human Health" associated with the informal treatment of WEEE with evidence of the impacts related to the individual process steps for 1 kg of e-plastics (1.86% cut-off).

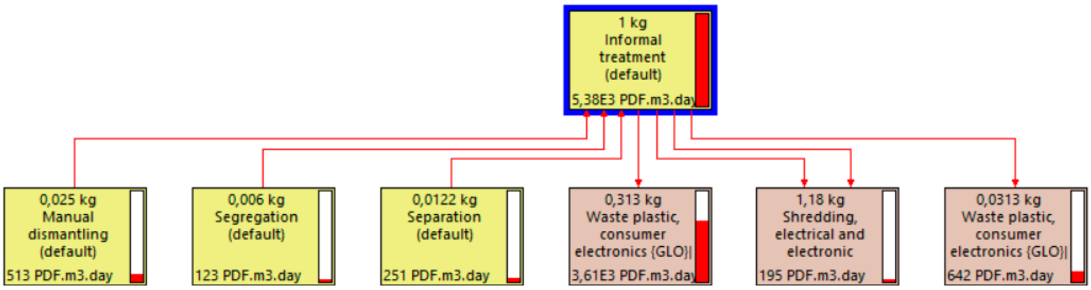

Figure S2. Network of the USEtox end-point results for the damage category "Ecosystems" associated with the informal treatment of WEEE with evidence of the impacts related to the individual process steps for 1 kg of e-plastics (1.86% cut-off).

Table S3. Environmental impacts associated with the five end-of-life scenarios (WEEEP-I, WEEEP-L, WEEEP-RD, WEEEP-RCS, WEEEP-RBP) for 1 kg of e-plastic residues (USEtox results for the impact categories "human toxicity, cancer"; human toxicity, non-cancer"; "freshwater ecotoxicity" (mid-point) and for the damage categories "human health" and "ecosystems" (end-point).

**Impact Categories**	**WEEEP-I**	**WEEEP-L**	**WEEEP-RC**	**WEEEP-RCS**	**WEEEP-RBP**
HT, cancer [CTU/kg]	5.17×10^{-8}	9.06×10^{-8}	2.72×10^{-8}	2.39×10^{-8}	1.67×10^{-8}
HT, non-cancer [CTU/kg]	2.50×10^{-6}	1.07×10^{-6}	6.75×10^{-7}	5.47×10^{-7}	1.47×10^{-7}
Freshwater ecotoxicity [PAF.m3.day]	23,091.59	41,037.82	8,172.20	6,733.497	2,305.77
**Damage Categories**					
Human health [DALY]	3.94×10^{-6}	7.47×10^{-6}	5.88×10^{-7}	2.13×10^{-6}	1.75×10^{-6}
Ecosystems [PAF.m3.day]	20,518.91	11,545.79	1,152.88	4,086.10	3,366.74

References

1. Pathak, P.; Srivastava, R.R.; Ojasvi. Assessment of legislation and practices for the sustainable management of waste electrical and electronic equipment in India. *Renew. Sustain. Energy Rev.* **2017**, *78*, 220–232.
2. Greenpeace. *Recycling of Electronic Wastes in China & India : Workplace & Environmental Contamination Electronic Wastes Contamination*; Greenpeace: Amsterdam, The Netherlands, 2005.
3. Bakhiyi, B.; Gravel, S.; Ceballos, D.; Flynn, M.A.; Zayed, J. Has the question of e-waste opened a Pandora's box? An overview of unpredictable issues and challenges. *Environ. Int.* **2018**, *110*, 173–192, doi:10.1016/j.envint.2017.10.021.
4. Gómez, M.; Peisino, L.E.; Kreiker, J.; Gaggino, R.; Cappelletti, A.L.; Martín, S.E.; Uberman, P.M.; Positieri, M.; Raggiotti, B.B. Stabilization of hazardous compounds from WEEE plastic: Development of a novel core-shell recycled plastic aggregate for use in building materials. *Constr. Build. Mater.* **2020**, *230*, doi:10.1016/j.conbuildmat.2019.116977.
5. Santhanam, N.; Ramesh, B.; Agarwal, S.G. Experimental investigation of bituminous pavement (VG30) using E-waste plastics for better strength and sustainable environment. *Mater. Today Proc.* **2020**, *22*, 1175–1180, doi:10.1016/j.matpr.2019.12.057.
6. Jullien, A.; Proust, C.; Yazoghli-Marzouk, O. LCA of alternative granular materials—Assessment of ecotoxicity and toxicty for road case studies. *Constr. Build. Mater.* **2019**, *227*, 116737, doi:10.1016/j.conbuildmat.2019.116737.
